# Selections

**Published:** 1892-05

**Authors:** 


### Selections.

### ABSTRACTS FROM "THE JOURNAL OF THE BRITISH DENTAL ASSOCIATION," MARCH, 1892.

THE following selections have been made from the letters of several correspondents in relation to Dr. Mitchell's paper published in the January number of this journal.—ED.]

"TO THE EDITOR OF 'THE JOURNAL OF THE BRITISH DENTAL ASSOCIATION:'

"SIR,—Mr. Smith Turner has omitted to mention that Mr. W. Mitchell, about whose offensive effusion in the INTERNATIONAL DENTAL JOURNAL he writes, is a member of the British Dental Association and of the Odontological Society of Great Britain. It is this fact alone, of course, which lends the least importance to Mr. Mitchell's writings. Under the circumstances, I think the members of both those bodies are justified in calling upon Mr. Mitchell either to substantiate or to withdraw and apologize for the statements which he has ventured to put forth. First, there are only three dental journals published in this country. Will Mr. Mitchell give us one citation from either of these papers in which the 'value of (respectable) American degrees is questioned and ridiculed'? Secondly, there are three dental schools in London,—the Dental Hospital, National Dental Hospital, and Guy's Hospital. Will Mr. Mitchell, after discussing the qualifications of the teachers and the method of instruction carried out, give us the grounds upon which he bases the judgment that these schools are 'more of a cheap burlesque than a worthy replica' of American colleges? Thirdly, the number of leading men in this country who hold American degrees could be represented by the fingers of one hand. Will Mr. Mitchell, in face of this fact, give us the evidence upon which he founds the statements that 'the best and most progressive students seek the halls of learning in America to secure that which they were unable to obtain here'?" . . . S.

"To the Editor of 'The Journal of the British Dental Association:'

"Sir,—There is a tone of viciousness about Mr. W. Mitchell's communication to the International Dental Journal which cannot be passed over in silence, and, for the honor of the school I represent, I unhesitatingly pronounce his remarks to be absolutely false in word and in spirit. Considering that in all branches of science and art students visit continental schools, is it to be wondered at if dental students should follow a similar course? It would be a direct insult to the body of students who have graduated at our colleges were I to qualify them under the comparisons of good, better, best; but I can say that of those who have distinguished themselves most in their academic career not one has sought the American diploma. . . . Mr. Mitchell's sympathies with American dental colleges may be well meant, but I fail to see the origin of his enthusiasm, and certainly, if he desires to uphold the spirit of the journal he supports with his communication to advocate *international* dental education, he will fail by his unjust strictures on the merits of the English schools. All lovers of science are proud to acknowledge the work done in schools of honorable reputation, and long may it be before that feeling dies out. Mr. W. Mitchell would have done himself more credit had he made himself familiar with the internal working of our schools before he penned his unfortunate communication; as it is, I can only regard his remarks as the result of ignorance or personal pique. Apologizing for trespassing upon your valuable space,

"Yours very truly,

"F. Henri Weiss,

"*Dean of the National Dental College.*"

---

"To the Editor of 'The Journal of the British Dental Association:'

"Dear Sir,—In the last number of the *Journal*, Mr. Mitchell asks why the best English dental students go to America to complete their education?

"With some personal experience of English and American dental schools, I should say our best students stay at home, where they are quite as well instructed in technical knowledge and professional ethics.

"In short, Mr. Mitchell's assumption is, throughout, based on imagination.

"I am, dear sir,
"Yours faithfully,
"F. NEWLAND-PEDLEY, F.R.C.S., L.D.S.

"March 3, 1892."

---

"TO THE EDITOR OF 'THE JOURNAL OF THE BRITISH DENTAL ASSOCIATION:'

"SIR,—In an extract from an article by Mr. W. Mitchell, quoted by Mr. Smith Turner in your last issue, Mr. Mitchell asks, 'Why is it that the best and most progressive students, after completing their course on this side, and having learned all their instructors were capable of imparting to them here, seek the halls of learning in America?' etc.

"The answer is a very simple one. During the years I have been officially connected with the Dental Hospital of London, about two hundred and thirty students have been educated there, and received their diplomas from the Royal College of Surgeons; of these, not more than eight have gone to America, and certainly they were in no special sense, or indeed in any sense, the best and most progressive of our students. In one case, the student who came out top of the list of an American college at which he attended had been several times unsuccessful before obtaining his diploma in England.

"Mr. Mitchell, no doubt, will feel himself in honor bound to give similar publicity to the answer that he has already given to the question.

"MORTON SMALE,
"*Dean, Dental Hospital of London.*"

---

"TO THE EDITOR OF 'THE JOURNAL OF THE BRITISH DENTAL ASSOCIATION:'

"SIR,—On reading the extract from Mr. W. Mitchell's speech, with which you favored us in last month's *Journal*, my curiosity was awakened by the cock-sure audacity of his assertion, and I sought to test its accuracy by a reference to the 'Dentists' Register' for 1891.

"The result is as I expected. The statement is but another example of spread-eagleism, resulting from a limited knowledge and a too persistent and exclusive association with co-patriot log-rollers. Had Mr. W. Mitchell kept his eyes open, and in a desire for truth made honest use of the opportunities placed within his reach by the very people whom it is his pleasure to decry and revile, and had he at the same time read with intelligence the dental literature of his own country, I think he would have crowed less loud and struck a truer key in estimating the comparative merits of British and transatlantic education, and the efficiency of their products. That our *best* students have gone, or do go, to finish in America, I have no hesitation in denying, and in proof thereof have but to quote the names of Tomes, Mummery, Smith Turner, Gartrell, Underwood, Parkinson, Hepburn, Hutchinson, Woodhouse, Smale, Lloyd Williams, and a whole host of others who, in point of head or hand, will hold their own with the best that ever an American college turned out. As for the rank and file, I am convinced from a pretty extensive intercourse with dentists in Britain and in America that, taking them *all round*, the British dentist is better trained for his life's work, and therefore more reliable, than his transatlantic brother. . . . That our system of education is more than a 'cheap burlesque' of his vaunted Alma Mater is shown by the fact that in America at present the engrossing topic in dental circles of repute is how to raise the quality of the teaching in American schools so as to assimilate it to the British standard. . . . We have had *much* good from America, Mr. W. Mitchell, *but not* ALL. A great deal even of that *much* has been taken from the old country and re-shipped,—an old friend with a new face. . . .

"KOSMOS.

"March, 1892."

---

"TO THE EDITOR OF 'THE JOURNAL OF THE BRITISH DENTAL ASSOCIATION:'

"SIR,—What ungrateful wretches we European dentists must be! When all our questionable means to secure a diploma of one of our own colleges,—which, after all, we are told, is only, well, 'a cheap burlesque,'—has failed, Brother Jonathan receives us with open arms, and for a few dollars grants us a genuine bogus article, and yet we dare question its value. Surely, sir, as Mr. Turner says, 'Comment is unnecessary.' Yet I think one statement should not be allowed to pass unanswered. The best of English students generally *are* satisfied with the curriculum here, and do not seek the 'halls of learning in America;' and the few I have come in contact with who aspired to the American doctorate, have not proved themselves in practice to be in any way superior to their fellow-students who have been content with the teaching received in English schools.

"Yours truly,

"R. Edwards,

"*Dean of the Liverpool Dental Hospital.*

"Liverpool, March 4, 1892."

---

### ALCODIFORM[1] IN CAPPING NERVES.

If the nerve is very much exposed, apply a ten-per-cent. solution of muriate of cocaine. Cut away part of the nerve and bleed it freely. After hemorrhage has ceased, cover the nerve with a thin coating of alcodiform. As soon as the alcohol has evaporated, cover this with a thin layer of chloro-percha, and as soon as the chloroform evaporates, flow over the gutta-percha some cement. After this cement hardens, add more cement. After cutting away excess of cement the cavity is ready for permanent filling. The cavity must be kept dry with dam during this process. If you get too much alcodiform on the nerve it can be reduced by touching it with a piece of bibulous paper saturated with alcohol. When the nerve is very slightly exposed, bleed freely, apply alcodiform, and cover with chloro-percha, leaving off the cement if you fill with an amalgam or any plastic filling.

S. M. Johnson,

*Texas Dental Journal.*

---

[1] Iodoform with just enough alcohol to make a thick, creamy mass.

